# Special Issue “Novel Mechanisms of Bacterial Antibiotic Resistance and Strategies to Fight Them”

**DOI:** 10.3390/ijms27083432

**Published:** 2026-04-11

**Authors:** Valentina Straniero, Begoña Monterroso, Silvia Zorrilla

**Affiliations:** 1 Dipartimento di Scienze Farmaceutiche, Università Degli Studi di Milano, Via Luigi Mangiagalli, 25, 20133 Milano, Italy; 2 Department of Crystallography and Structural Biology, Instituto de Química Física Blas Cabrera, Consejo Superior de Investigaciones Científicas (CSIC), Serrano 119, 28006 Madrid, Spain; 3 Department of Molecular and Cellular Biosciences, Centro de Investigaciones Biológicas Margarita Salas, Consejo Superior de Investigaciones Científicas (CSIC), Ramiro de Maeztu 9, 28040 Madrid, Spain

Antimicrobial resistance (AMR) is nowadays widely recognized as one of the most challenging problems in human health, with deep impact also in animals and the environment, highlighting the interconnected vulnerabilities shared across the One Health spectrum [1]. Innovative strategies are necessary to tackle this public health burden, ideally from multiple angles, to reach solutions circumventing the several mechanisms pathogenic bacteria have developed to withstand current treatments. As shown in this Special Issue, this joint effort of the scientific community can cover very diverse approaches, such as the development of new small antimicrobial molecules [2,3], the repurposing of existing ones [4], further exploration of promising alternatives, such as phagotherapy [5], and the detection and characterization of harmful bacterial populations in potential sources of infection that are commonly overlooked [6].

This should parallel with research programs centered on better understanding how bacteria orchestrate their life functions, how they are tuned to maximize their chances to survive, and the key role of the bacterial membrane, aspects also covered within this Special Issue [3,4,7]. Even more enigmatic are the chemical signaling systems bacteria use to coordinate collective behaviors, including virulence [7]. Deciphering these cellular pathways may unveil new targets spanning from individual proteins to macromolecular complexes or emergent properties.

Bottom-up synthetic biology approaches involving reconstitution of bacterial modules in cytomimetic systems of increasing complexity have proven useful for understanding how these modules operate and are regulated, since the small size of bacteria hampers direct in vivo examination [8]. Macromolecular crowding, membranes, microenvironments, and compartments are among the relevant features to be considered in these cell-like systems [9], as they can largely impact interactions, spatial distribution, and arrangement of molecules [10] in bacterial contexts [11]. Indeed, biochemical reconstitution in crowding systems has been used to assess the mechanisms used by drugs killing bacteria in combination with in vivo analyses, as shown in this Special Issue [2] and elsewhere [12]. Additional applications of synthetic biology in the field include engineering phages, for example, to degrade biofilms (see [13] and references therein), or modification of microbes or mammalian cells for antibacterial therapy [14].

Like in many other fields, also in AMR research, the use of artificial intelligence (AI) is growing exponentially, finding multiple applications in diagnostics, resistance prediction, and antibiotic discovery [15,16], enhancing the performance of well-established technologies as reported in this Special Issue [17]. Remarkably, AI can be used synergistically with synthetic biology, among other approaches, in the quest for innovative antimicrobials [18,19].

The review by Bravo, Espinosa, and coworkers is focused on the *One Earth* concept [7] that the authors previously introduced as a holistic approach to deal with the increasing burden of antimicrobial resistance [20]. In contrast to more classical schemes emphasizing the need to kill harmful bacteria to tackle AMR, this thoughtful view proposes finding an equilibrium with the bacterial world [21]. Implementation of this conception requires a deep understanding of the multiple pathways involved in antimicrobial resistance to identify ways to reduce the synthesis of virulence factors. A remarkable advantage of this approach is the preservation of biodiversity, widely recognized to be related to climate change [22], thus reducing the impact of antimicrobials on one of the foremost global societal challenges.

After nicely describing the problem of AMR and illustrating how current bactericidal interventions have inexorably resulted in the selection of superbugs, they introduce the global alternative they conceived, aligned with the lessons of Louis Pasteur [7]. The main body of the article delves into the key players in AMR: the resistome [23], the mobilome [24], and the nichome [25] (Figure 1), which Espinosa and coworkers authoritatively analyze, stressing that AMR is the result of multiple mechanisms rather than a single one.

The different modes of action of currently available antibiotics are described and classified according to the essential cellular process they interfere with: cell wall synthesis, membrane integrity, synthesis of DNA/RNA, proteins, or metabolites. Examples of specific antibiotics, their targets, and the basis of resistance are presented, and the authors feature the usefulness of cutting-edge technologies, including AI, to find new antibiotics [26]. Acknowledging the fact that the most frequent source of AMR is the acquisition of genes encoded by mobile genetic elements, the authors dedicate an important part of their article to analyzing the various pathways through which horizontal gene transfer occurs, critically evaluating possible strategies to interfere with them as a means to curb bacterial infections. They point out that universal inhibition of gene transfer would have an important drawback, that is, the decrease in bacterial genetic diversity [7]. They also remark that a very marginal percentage of the transferred genes are antibiotic resistance ones, while others, conferring adaptive advantages, for example, those facilitating colonization of new niches, are more frequently mobilized [7]. This connects with the last part of the review, focused on the nichome. The behavior of bacteria as communities that either compete or cooperate [27] and the implications of cell signaling and biofilm formation in the context of infection are discussed. The authors highlight the importance of transcriptional regulatory factors, particularly nucleoid-associated proteins, in the orchestration of gene expression, crucial for adaptation to new niches. Actually, some of these proteins have recently been described to enhance bacterial fitness and resistance to stresses such as antibiotic treatment through the formation of biomolecular condensates [11,28]. These dynamic structures are often promoted by multivalent protein-DNA interactions and by macromolecular crowding in the bacterial cytoplasm, especially in the nucleoid region [11]. Attention is called to the modulation of the affinity of transcriptional regulatory factors for their DNA operators through posttranslational modifications like phosphorylation, still poorly explored in bacteria [28,29].

The final reflection of the authors remarks the need for understanding how bacteria living in natural communities behave, communicate, and develop resistance to antimicrobials to devise strategies to reduce virulence while keeping biodiversity as a means to preserve human health with minimal impact on the entire ecosystem.

The review by Chiarelli et al. explores iron acquisition and metabolism as innovative targets to combat AMR [3]. Traditional antibiotics impose strong selective pressure, accelerating resistance development, whereas anti-virulence strategies aim to disarm pathogens without killing them, reducing this pressure [30,31]. This is in line with the *One Earth* philosophy previously described (see above and [7]).

Iron is essential for bacterial survival, acting as a cofactor in key enzymatic processes. Its bioavailability in the host is limited due to nutritional immunity [32]. Pathogens overcome this through sophisticated uptake systems, including siderophores, high-affinity iron-chelating molecules, and heme acquisition mechanisms [33,34,35]. These unique bacterial pathways, absent in humans, offer selective targets for novel therapeutics.

The review focuses on four main strategies:

1. Iron Depletion: Chelators such as DIBI, a polymeric 3-hydroxypyridin-4-one (Figure 2a), demonstrated activity against *Staphylococcus aureus* (including MRSA) and *Acinetobacter baumannii*, with reduced toxicity compared to traditional chelators [36,37,38,39,40].
2. Iron Mimetics: Gallium (Ga^3+^), which mimics Fe^3+^ but cannot undergo redox reactions, disrupts iron-dependent enzymes and siderophore-mediated uptake. Gallium compounds, including Ga-protoporphyrin IX (Figure 2b), showed efficacy against ESKAPE pathogens (*Enterococcus faecium*, *S. aureus*, *Klebsiella pneumoniae*, *A. baumannii*, *Pseudomonas aeruginosa*, and *Enterobacter* spp.) and resistant strains, though bioavailability and nephrotoxicity remain challenges [41,42,43,44,45,46]. Nanocarrier-based delivery systems and hydrogels are being developed to improve local administration and reduce side effects [47,48,49,50,51].
3. Inhibition of Siderophore Biosynthesis: In *Mycobacterium tuberculosis*, inhibitors targeting MbtA and MbtI enzymes have shown promising results, with 5′-*O*-*N*-salicylsulfamoyl adenosine (Sal-AMS) (Figure 2c) analogs and furan-based derivatives reducing siderophore production and bacterial growth [52,53,54,55,56,57,58,59,60,61]. Similarly, in *P. aeruginosa*, pyoverdine inhibitors such as 5-fluorocytosine and PvdQ-targeting compounds significantly attenuate virulence [62,63,64,65,66].
4. Trojan Horse Strategy: Exploiting siderophore uptake systems to deliver antibiotics enhances intracellular penetration. Cefiderocol (Figure 2d), a catecholate siderophore–cephalosporin conjugate, has received FDA approval and demonstrated potent activity against carbapenem-resistant Gram-negative pathogens in clinical trials [67,68,69,70,71,72,73]. Artificial siderophores like the 1,3,5-*N*,*N*′,*N*″-Tris-(2,3-dihydroxybenzoyl)-triaminomethylbenzene (MECAM) further expand this approach, enabling conjugation with diverse antibiotics for broad-spectrum or targeted activity [74].

Iron metabolism represents a multifaceted opportunity for anti-virulence therapy, combining pathogen-specific targeting with reduced resistance pressure. However, challenges remain in optimizing pharmacokinetics, delivery systems, and minimizing toxicity. Continued research into siderophore biology and iron homeostasis will be critical for developing next-generation antimicrobials [46,48,49,75].

The review by Molina et al. comprehensively describes a substantial number of the bacterial defense systems described to date [5], whose knowledge could be instrumental for the development of phage-based antimicrobial therapies. By classifying them according to the immunity strategy adopted by the bacteria (degradation of the genetic material of the bacteriophage, triggering of an abortive infection, and inhibition of genome replication; Figure 3), they provide an illustrative roadmap for these diverse antiphage mechanisms [76].

These immunity strategies correlate with the stage of the infection cycle [77] and involve, in turn, several different systems. Degradation of phage nucleic acids, the most prevalent defense system in the microbial realm, is at the core of restriction-modification (RM) systems, CRISPR-Cas systems, the Gabija system, and nucleic-acid sequence-independent systems. RM systems, grouped in four categories depending on their characteristics, are prevalent and general immune mechanisms in prokaryotes [78] and involve the cleavage of nucleic acids from invading phages based on epigenetic modifications [79]. Other RM systems relying on different mechanisms to discriminate between self and non-self are the RM-like defense systems, the bacteriophage exclusion (BREX) system, and the defense island associated with the RM (DISARM) system. CRISPR-Cas systems, with different classes and types based on the genes encoded and the nature of their effector [80] (Figure 3), are among the most prevalent defenses, acting through interference with phage nucleic acids through an adaptive immune response enabled by past infections. The Gabija system is a protein complex with endonuclease activity that nicks double-stranded DNA [81].

Abortive infection (Abi) systems function by inducing cell death before the maturation of the phage progeny upon the recognition of infection, halting phage spread among cells. Several of these mechanisms are based on the infection-triggered production of a signaling molecule that activates a cell-killing effector [82] through different mechanisms that define their classification, such as membrane impairment and DNA degradation (CBASS systems) or depletion of cellular nicotinamide adenine dinucleotide (pyrimidine cyclase system for antiphage resistance–Pycsar–systems), depletion of NAD+ in the cell (Thoeris system), and growth and infection arrest (type III CRISPR-Cas systems). Retron systems, through a still unknown mechanism, rely on nucleoprotein complexes to assess for bacterial cell machinery integrity that, if affected, leads to cell death [83] (Figure 3). Also unknown is the mechanism by which the Lamassu family, involving structural maintenance of the chromosomes (SMC) proteins, triggers abortive infection [84]. Activation upon phage infection of toxin-antitoxin systems results in cell death or growth arrest by degradation of the antitoxin, counteracting the toxin that targets essential cellular processes [85], while with the pore-forming proteins, gasdermins lead to the disruption of membrane integrity.

*Prokaryotic argonautes* (pAgo) are ubiquitous proteins with a defense role that depends on their catalytic or non-catalytic nature. While the first ones induce the degradation of the phage genetic material, the latter type participates in immunity by abortive infection [86].

Finally, among the mechanisms directly targeting phage DNA or RNA synthesis, hence hindering viral replication, are the chemical defense systems and the depletion of DNA or RNA mechanisms. The production of small molecules, such as anthracyclines or aminoglycosides, or RNA chain terminator molecules, inhibits viral replication by affecting the synthesis of phage nucleic acids (Figure 3). Infection-triggered defensive enzymes eliminate DNA nucleotides essential for replication.

This thorough revision of bacterial defense systems shows their great potential for future biomedical applications based on the knowledge of the molecular mechanisms involved and has wide implications for phage-based therapies currently under development.

The article by Massidda, Straniero, and coworkers [2] targets *Streptococcus pneumoniae*, which is listed among the World Health Organization’s priority pathogens for which new antibiotics are urgently needed [87]. Traditional antibiotic development has stagnated, and most new drugs are analogs of existing classes [88,89,90]. This scenario underscores the need for innovative approaches targeting essential bacterial processes, such as cell division, which is orchestrated by the highly conserved protein FtsZ. FtsZ is a tubulin homolog that forms the Z-ring and recruits divisome components to complete cytokinesis [11,91]. Inhibiting FtsZ disrupts bacterial proliferation and ultimately leads to cell death, making it an attractive target for next-generation antimicrobials [92,93,94].

In this study, the authors presented advances in this field by demonstrating that benzodioxane–benzamide derivatives, previously active against *S. aureus* and *Bacillus subtilis* [95,96], also exhibit potent activity against *S. pneumoniae*. Four compounds, FZ95, FZ100, FZ116, and FZ118 in Figure 4a, showed promising minimal inhibitory concentrations (MICs) and displayed bactericidal effects within 180 min of exposure. Notably, FZ116 induced rapid bacteriolysis, with morphological changes evident after only 15 min, suggesting that specific structural features may enhance potency and influence additional cellular processes (Figure 4b).

Microscopy revealed phenotypes consistent with FtsZ inhibition: enlargement, bulging, and eventual lysis, indicative of a simultaneous block in sidewall elongation and division [97]. In vitro assays under crowding cytomimetic conditions confirmed that these compounds hyperstabilize FtsZ polymers, prolonging their lifetime and altering depolymerization profiles. These findings align with previous observations in *E. coli* [12], supporting a conserved mechanism of action across species.

An initial Structure–Activity Relationship (SAR) analysis highlights the importance of linker length and stereochemistry. Compounds with ethylenoxy linkers were significantly more active than those with methylenoxy linkers, and the *erythro* isomers exhibited markedly superior performance compared to their *threo* counterparts, demonstrating that stereochemical configuration is crucial for effective target binding [96,98]. Such insights pave the way for rational optimization of this chemical class. While the absence of a crystal structure for pneumococcal FtsZ remains a limitation, docking studies in related species suggest a conserved binding pocket that could guide future design efforts.

This work demonstrates that targeting bacterial cell division through FtsZ inhibition is a viable strategy to combat AMR and that benzodioxane–benzamides represent a promising scaffold for broad-spectrum agents, active against pathogens with diverse morphologies.

Also centered on infections caused by the important respiratory pathogen *S. pneumoniae* is the article by Maestro, Sanz, and coworkers, where they describe a powerful drug repurposing approach [4]. Specifically, they screened the Prestwick^®^ Chemical Library, comprising over a thousand molecules with a variety of mechanisms of action. The authors obtained more than 150 initial hits that reduce cell growth, according to turbidity assays conducted on planktonic cultures of the pathogen. After discarding already known antimicrobials with antistreptococcal activity, around 30 molecules were selected for thorough analysis at lower doses, and a few chemicals were finally identified as able to drastically decrease bacterial viability.

The authors then undertook a careful characterization of 7 hits by determining their minimal inhibitory concentrations (MICs) on various strains and gaining insight into their molecular mechanisms of action. MICs between ~10 and ~55 µg/mL were obtained, in the range of those published for other antimicrobials [2,99,100,101]. By examining the structures of the compounds under investigation, the authors identified noteworthy common features, including marked hydrophobicity and the presence of an amine functionality that is expected to be protonated at physiological pH (Figure 5a). In light of such observations, they hypothesized that these amphiphilic molecules could alter the bacterial membrane by establishing both electrostatic and hydrophobic interactions with the polar head of the phospholipids and the hydrophobic fatty acid chains. To explore this option, membrane permeabilization assays were conducted using a fluorescent probe that interacts with nucleic acids, as a result of which its fluorescence emission is dramatically enhanced. The authors conclude that, except for one compound with a distinct structure, the selected drugs actually compromise membrane integrity, allowing internalization of the fluorophore (Figure 5b), thus confirming their hypothesis [4]. Moreover, the correlation between MICs and permeabilization degree for the different molecules further strengthens the idea that alterations in the membrane are part of the killing mechanisms of these drugs.

Ortiz-Miravalles et al. envision that the seven selected compounds could represent promising candidates for repurposing as antibiotics, serve as lead molecules for the development of new antimicrobials, or be co-administered synergistically with currently available drugs targeting *S. pneumoniae*. Interestingly, some of the molecules identified are classified as ion channel blockers, described to inhibit efflux pumps in other instances, although this activity does not seem to be sufficient for significant antipneumococcal capability [4]. Finally, the bactericidal action of the reported molecules, mediated by non-specific membrane distortion, may render them less susceptible to resistance, while additional pathways could also contribute to the overall activity, as reported for other drugs acting at the membrane level.

Lenart-Boroń and cols. identify pathogens infecting skin wounds of companion animals and assess their antibiotic resistance and the presence of antibiotic resistance genes (ARGs; [6]). Both factors constitute a health risk because of the possibility of interspecies transmission by infection of pet owners or veterinarians with antibiotic-resistant bacteria (ARB) and transfer of ARGs to the human microbiota [102], as most bacterial pathogens in companion animals usually occur in humans. Additionally, wound treatment in veterinary medicine relies on the use of broad-spectrum antibiotics commonly used in human medicine, further contributing to the current antimicrobial resistance problem [103].

Among the 136 bacterial strains isolated from dogs, cats, and rabbits, Gram-positives and Gram-negatives were essentially equally present, being the dominant genera—as identified through chromogenic media and MALDI-TOF spectrometry—*Staphylococcus* (*S. pseudintermedius* and *S. aureus*), *Enterococcus* (*E. faecalis*), *E. coli*, *Acinetobacter* (*A. ursingii*), and *Pseudomonas* (*P. aeruginosa*). Antimicrobial resistance tests were conducted on a substantial number of bacterial isolates against 16 antibacterial agents in five combinations selected according to the target organism. Results evidenced different prevalences of antibiotic resistance depending on the genera and the antimicrobial, and, in some cases, none of the antimicrobials tested was effective against all strains within a genus (Figure 6). Notably, *Enterobacterales* were resistant to β-lactam antibiotics in a high percentage, while all *Pseudomonas* isolates were not vulnerable to several antimicrobials of different families. In this regard, authors stress the importance of resistance against last-resort antimicrobials in microorganisms that can be easily transmitted between animals and humans [104], highlighting their frequent finding of insusceptibility to imipenem (carbapenem antimicrobial) and resistance to this antimicrobial in all *Enterococcus* and *Pseudomonas* spp. isolates.

The authors conducted PCR tests for the identification of 12 genetic determinants of antibiotic resistance against all antimicrobial classes employed in the treatment of Gram-positive and Gram-negative bacteria. The genes examined were common for Gram-positive and Gram-negative bacteria when the associated resistance mechanism was maintained, while others were assessed only for one of these two bacterial groups when the resistance mechanism was specific. The most frequently detected resistance gene was *strA* (resistance to streptomycin), followed by *sul3* (resistance to sulfonamide) in both Gram-positive and Gram-negative isolates, and an extended-spectrum beta-lactamase (ESBL) determinant (*blaTEM*) in Gram-negative bacteria.

The study reflects the great variability in the composition of bacterial species that can be found in pets’ wounds, and that their prevalence, even in those with higher occurrence, is not high enough to foresee their presence. The observed prevalence of multidrug-resistant bacteria and the detection of bacteria resistant to the majority of antimicrobials tested that are also human pathogens strongly point towards the probable future scenario of the veterinary field needing new antimicrobial therapies.

The article by López-Cortés and coworkers deals with the detection of AMR by combining a well-consolidated technique, mass spectrometry (MS), with innovative AI approaches [17]. Specifically, they considered data obtained with the non-mainstream VITEK^®^ MS instruments and evaluated the capability of predicting AMR in three bacterial species: *E. coli*, *K. pneumoniae*, and *S. aureus*. Different models were applied to over two thousand spectra, finding important advantages for the classification algorithm CatBoost, which employs a gradient-boosting technique. The study also addresses the efficacy of transfer learning of models trained with data recorded by more conventional instruments from Bruker, finding suboptimal performance. The authors conclude that AI-powered evaluation of MS determinations is a promising tool to combat AMR, suggesting that standardization of spectrum acquisition and preprocessing procedures would facilitate its broad application.

In conclusion, this Special Issue includes review articles and original research manuscripts providing a variety of interesting concepts, ideas, results, and methodologies to advance the field of antimicrobial resistance. Thus, non-conventional views such as the *One Earth* philosophy are comprehensively described, as well as strategies based on iron-modifiers somehow aligned with this view. In addition, a panoply of the increasingly numerous molecular mechanisms within bacterial defense systems is scrutinized as potential targets for new antimicrobials, including phage-based therapies. Promising molecules altering membrane permeability are uncovered by repurposing, the antibiotic-resistant bacterial populations in animal wounds are discussed as an additional source of AMR transmission, and machine learning is shown to be useful in the field when combined with widely used analytical techniques. Finally, approaches are described exploiting key proteins involved in essential survival processes, such as cell division, as targets of small molecules. Despite years of research, the operation of the machinery responsible for chromosome duplication, segregation, and cell division remains far from well understood. Close inspection of these systems has revealed many new targets worth exploring, including the scaffold protein of the divisome, but also its interactions with regulatory factors and emergent properties such as phase separation into biomolecular condensates [8]. The latter are intriguing structures showing increasing connections with stress resistance and potentially involved in the development of persister cells tolerant to antibiotics, ultimately facilitating the acquisition of resistance [11,28,105]. Recent studies have unveiled the role of phase separation in the mechanisms used by antimicrobial peptides [106,107] and the ability of chemicals to alter the properties of biomolecular condensates assembled by cell division proteins [108], further supporting the idea that phase separation phenomena may open new horizons in AMR research.

## Figures and Tables

**Figure 1:**
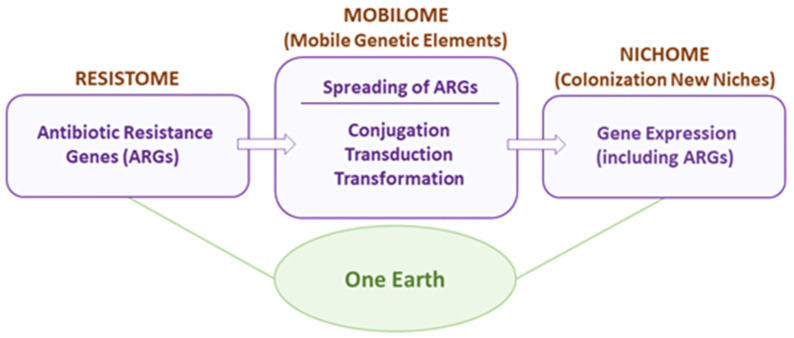
The *One Earth* concept as described in [7]. Figure taken from [7].

**Figure 2:**
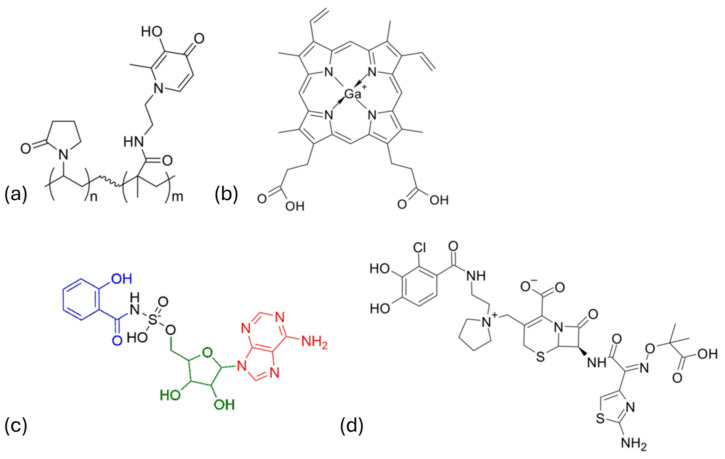
Chemical structure of representative compounds of the four strategies proposed in the review by Chiarelli and colleagues [3]: 3-hydroxypyridin-4-one DIBI (**a**); gallium protoporphyrin IX (**b**); 5′-*O*-*N*-salicylsulfamoyl adenosine (**c**); the salicylate moiety is colored in blue, the sugar in green, and the nucleobase in red); cefiderocol (**d**). Figure adapted from [3].

**Figure 3:**
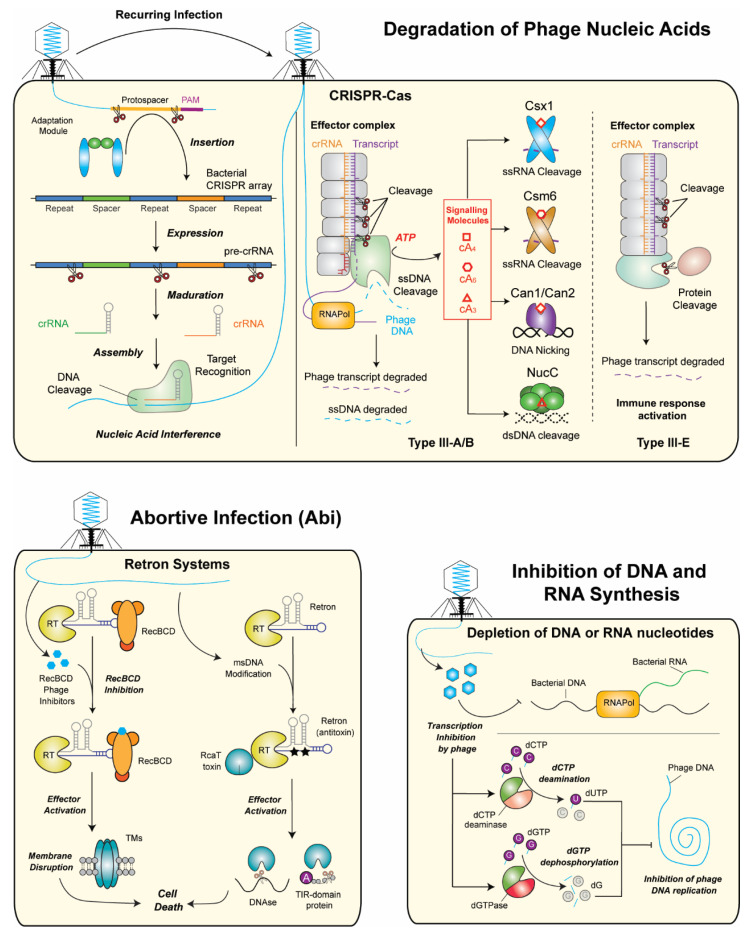
Examples of systems acting through the three immunity strategies adopted by the bacteria against infecting bacteriophages. Adapted from [5].

**Figure 4:**
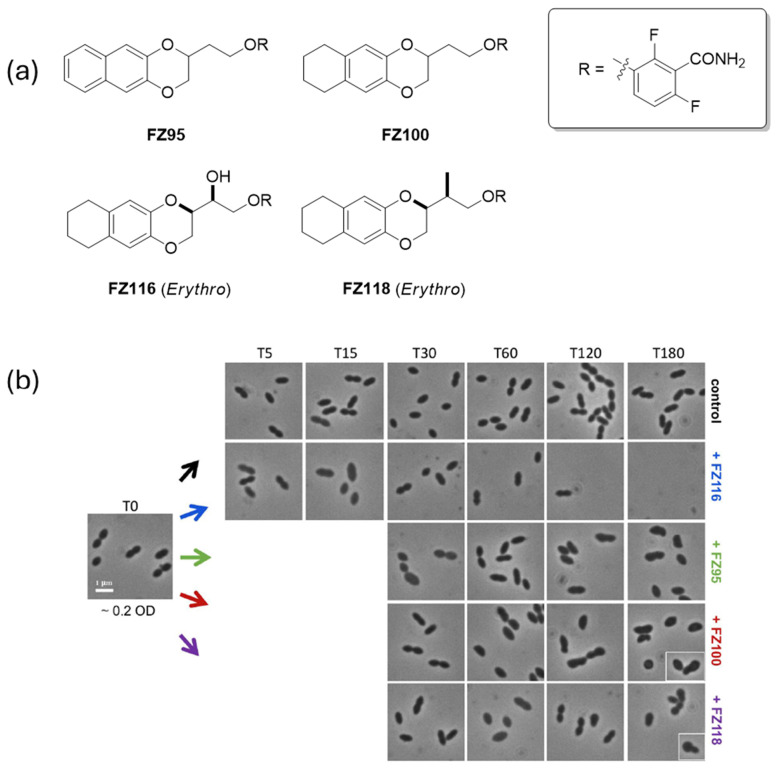
Benzodioxane–benzamide derivatives active towards *S. pneumoniae* (**a**) and their effects on *S. pneumoniae* morphology, by evaluation using phase-contrast microscopy at different time points (**b**). Figure adapted from [2].

**Figure 5:**
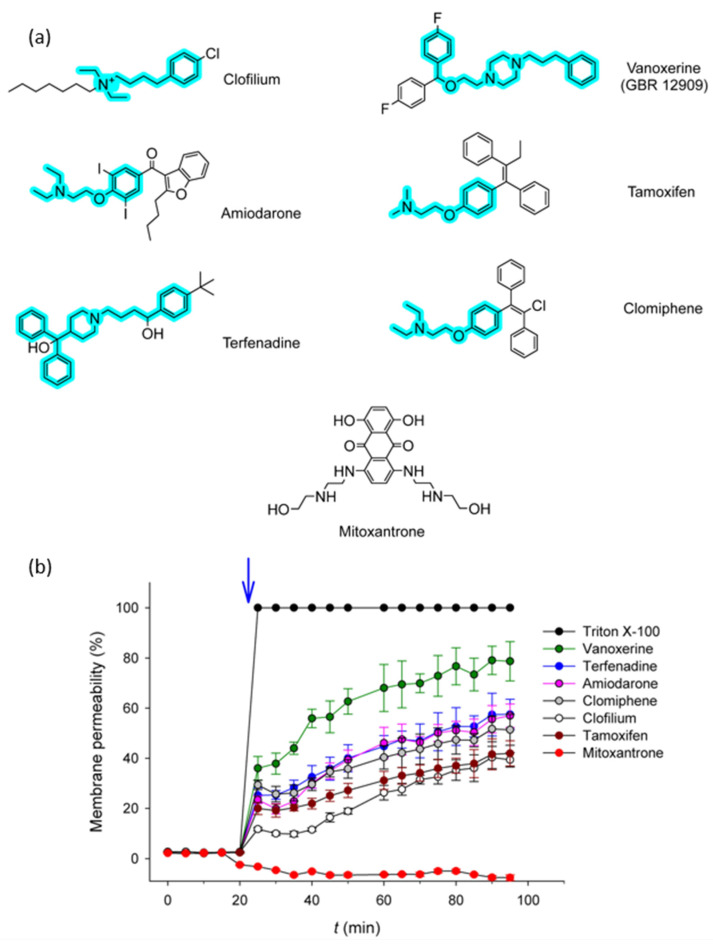
Molecules strongly affecting pneumococcal viability (**a**) and their impact on membrane permeability (**b**). Fragment common to the structures highlighted in cyan in (**a**) and time of addition indicated by an arrow in (**b**). Figure adapted from [4].

**Figure 6:**
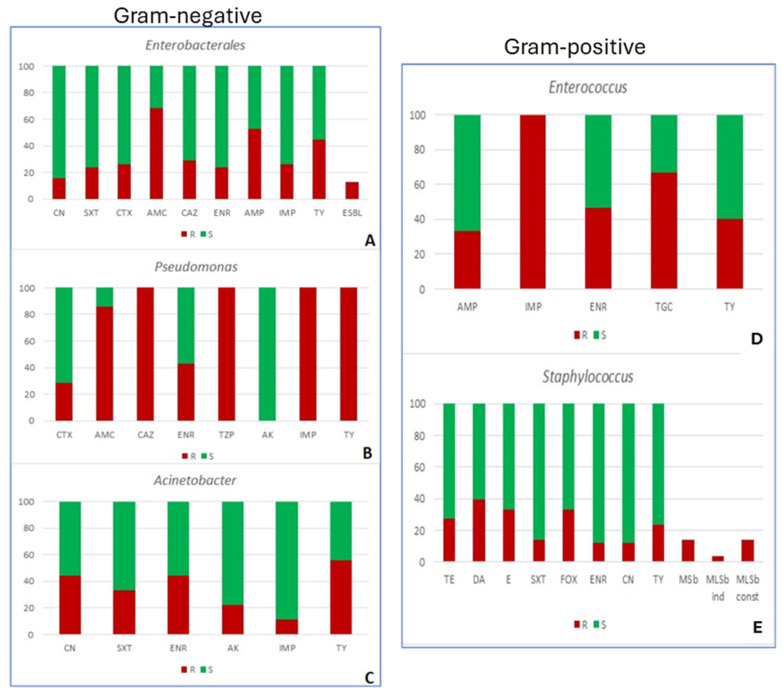
Share (%) of resistant (R) and susceptible (S) Gram-negative (**left**) and Gram-positive (**right**) bacterial strains isolated from wounds of companion animals. (**A**), *Enterobacterales* (**B**), *Pseudomonas* (**C**), *Acinetobacter* (**D**), *Enterococcus* (**E**), and *Staphylococcus*. In alphabetical order: AK, amikacin; AMC, amoxicillin/clavulanic acid; AMP, ampicillin; CAZ, ceftazidime; CN, gentamycin; CTX, cefotaxime; DA, clindamycin; E, erythromycin; ENR, enrofloxacin; ESBL, extended-spectrum-betalactamase-producing strains of Enterobacterales; FOX, cefoxitin; IMP, imipenem; MSb, resistance mechanisms to macrolides and streptogramins b; MLSb const, constitutive mechanisms of resistance to macrolides, lincosamids, and streptogramins b; MLSb ind, inducible mechanisms of resistance to macrolides, lincosamids, and streptogramins b; SXT, trimethoprim/sulfamethoxazole; TE, tetracycline; TGC, tigecycline; TY, tylosin; TZP, piperacillin/tazobactam. Taken from [6].
